# Research Progress and Prospects of Flavonoids in the Treatment of Hyperlipidemia: A Narrative Review

**DOI:** 10.3390/molecules30153103

**Published:** 2025-07-24

**Authors:** Xingtong Chen, Jinbiao Yang, Yunyue Zhou, Qiao Wang, Shuang Xue, Yukun Zhang, Wenying Niu

**Affiliations:** School of Basic Medical Sciences, Heilongjiang University of Chinese Medicine, Harbin 150040, China; chenxingtong@hljucm.edu.cn (X.C.);

**Keywords:** flavonoids, hyperlipidemia, research progress, lipid metabolism

## Abstract

Hyperlipidemia (HLP) is a disorder of human lipid metabolism or transport, primarily characterized by abnormally elevated levels of total cholesterol (TC), triglycerides (TGs), and low-density lipoprotein cholesterol (LDL-C) in the blood. It is a key factor contributing to the development of non-alcoholic fatty liver disease, obesity, diabetes, atherosclerosis, and cardiovascular and cerebrovascular diseases. Statistics show that the prevalence of dyslipidemia among Chinese adults is as high as 35.6%, and it has shown a trend of younger onset in recent years, posing a serious threat to public health. Therefore, the prevention and treatment of dyslipidemia carry significant social significance. The pathogenesis of hyperlipidemia is complex and diverse, and currently used medications are often accompanied by side effects during treatment, making the research and development of new therapeutic approaches a current focus. Numerous studies have shown that flavonoids, which are abundant in most medicinal plants, fruits, and vegetables, exert effects on regulating lipid homeostasis and treating hyperlipidemia through a multi-target mechanism. These compounds have demonstrated significant effects in inhibiting lipid synthesis, blocking lipid absorption, promoting cholesterol uptake, enhancing reverse cholesterol transport, and suppressing oxidative stress, inflammation, and intestinal microbiota disorders. This article reviews the latest progress in the mechanisms of flavonoids in the treatment of hyperlipidemia, providing a theoretical basis for future research on drugs for hyperlipidemia.

## 1. Introduction

Hyperlipidemia is a disorder of lipid metabolism, clinically characterized by dyslipidemia, including elevated levels of total cholesterol (TC), triglycerides (TGs), and low-density lipoprotein cholesterol (LDL-C) or decreased high-density lipoprotein cholesterol (HDL-C). It serves as a risk factor for obesity, non-alcoholic fatty liver disease, and cardiovascular and cerebrovascular diseases such as atherosclerosis [1]. Current clinical medications for hyperlipidemia mainly include statins, fibrates, ezetimibe, PCSK9 inhibitors, and bile acid sequestrants [2]. Although these drugs effectively reduce lipid levels, they are often associated with side effects. For example, statins may cause myotoxicity and hepatotoxicity [3], while fibrates can lead to liver function deterioration and increased creatinine levels [2]. Due to the numerous adverse reactions associated with these drugs, research on natural products has received significant attention. A growing body of studies has demonstrated that traditional Chinese medicines and their extracts, as well as dietary flavonoids and their derivatives, hold great value and potential in the treatment of hyperlipidemia [4]. This paper is a narrative review that aims to summarize the research progress on the mechanism of flavonoids in the treatment of hyperlipidemia by reviewing the relevant literature.

Current research has gaps in mechanisms: for instance, the specific mechanisms of flavonoids in hyperlipidemia subtypes remain unclear, and the exploration of new targets such as circRNA is insufficient. Future studies should focus on these directions and promote clinical trials to clarify their clinical positioning in hyperlipidemia.

## 2. Flavonoids

In recent years, increasing attention has been paid to the research and application of traditional Chinese medicines, their extracts, and active ingredients in diets. Studies have shown that natural products in many traditional Chinese medicines, fruits, and vegetables can regulate lipids through multiple pathways, and there is ample evidence confirming that certain natural compounds have therapeutic potential for diseases related to hyperlipidemia [5]. These chemical substances offer obvious advantages, such as defined components, easy preparation, and relatively clear toxicity and side effects, making them important targets for studying lipid metabolism regulation. Numerous studies have found that traditional Chinese medicines, fruits, and vegetables contain a rich variety of flavonoids [6,7,8,9,10], which have obvious regulatory effects on lipid metabolism [11]. Flavonoids are a class of natural secondary metabolites widely present in TCMs, with multiple biological activities. They have a 2-phenylchromone structure and can be divided into seven main types based on the degree of oxidation of ring C (central three-carbon chain), ring connection patterns, and different substituents in their nuclear structure. These types include flavones, flavonols, dihydroflavones, isoflavones, flavanols, chalcones, and anthocyanidins [12]. Most flavonoids exhibit lipid-lowering effects, and the representative flavonoids with lipid-lowering activities in their main types will be described in detail below.

### 2.1. Flavones

Flavones, including apigenin, luteolin, chrysin, acacetin, baicalein, robinin, and chrysoeriol, have been extensively studied. Among these, apigenin and luteolin stand out for their pronounced lipid-lowering properties. Apigenin is a natural flavonoid compound widely present in *Leonurus japonicus*, olive leaves, chamomile, parsley, celery, *Basella alba* L., artichoke, etc. [13,14,15,16]. Structurally, its A-ring bears hydroxyl (-OH) substituents at the 5- and 7-positions, while the B-ring harbors a hydroxyl group at the 4’-position. These hydroxyl moieties endow apigenin with high polarity, facilitating interactions with water molecules and other polar entities. More importantly, they serve as critical functional groups underlying its multifaceted biological activities. Emerging evidence has highlighted apigenin’s diverse properties, including anti-inflammatory [17], antioxidant [18], anticancer [19], and anti-apoptotic properties [20] and its ability to reduce lipid levels and insulin resistance [21].

Luteolin: Luteolin is present in *Taraxacum mongolicum*, *Chrysanthemum morifolium*, lotus stems, *Lonicera japonica*, cabbage, celery, apples, oranges, pomegranates, lemons, etc. [22,23,24,25,26,27]. Its structure features multiple hydroxyl groups, which serve as critical functional moieties for antioxidant and other biological activities. Emerging research has revealed its diverse bioactivities, including anticancer effects [28], lipid-lowering and anti-inflammatory properties [29], modulation of intestinal microbiota [30], and immunoregulatory functions [31]. These attributes endow luteolin with promising potential for drug development and functional food applications.

### 2.2. Flavonols

Flavonol compounds comprise quercetin, kaempferol, morin, galangin, myricetin, rutin, isorhamnetin, etc., with quercetin and kaempferol being most representative for their lipid-lowering properties. Quercetin: Quercetin is commonly found in traditional Chinese medicines such as *Astragalus membranaceus*, *Trichosanthes kirilowii*, *Albizia julibrissin* flowers, and *Morus alba* leaves, as well as in fruits and vegetables including grapes, onions, carrots, and potatoes [32,33,34,35,36,37,38,39,40]. Its basic nucleus is phenyl benzoyl ketone, forming a C6-C3-C6 structure where two benzene rings (A and B) are linked by an oxygen-containing pyran ring (C ring). The planar configuration of the three-ring system confers relative molecular polarity, enabling diverse biochemical interactions. As a pivotal flavonol, Quercetin exhibits multifaceted bioactivities: ameliorating metabolic syndrome [41], antibacterial effects [42], neuroprotection [43], anti-ferroptotic activity [44], hypoglycemic and anti-obesity properties [45], and antirheumatic effects [46]. It also plays a critical role in chemotherapy by mitigating the adverse effects of cisplatin, daunorubicin, and tert-butyl hydroperoxide [47]. Furthermore, quercetin demonstrates synergistic effects with various compounds and drugs, such as menadione and amoxicillin [48]. Owing to its diverse biological activities and therapeutic potential, quercetin is widely recognized as one of the most significant flavonols in dietary sources and pharmaceutical research.

Kaempferol: It has been reported that kaempferol is present in *Ginkgo biloba* leaves, *Carthamus tinctorius*, *Cnidium monnieri*, blueberry leaves, figs, legumes, cauliflower, cabbage, strawberries, tea, and tomatoes [49,50,51,52,53,54,55]. Its core structure features the typical tricyclic system of flavonols, with two benzene rings (A and B rings) linked by a central three-carbon chain (C ring) to form a C6-C3-C6 skeleton. Kaempferol exhibits diverse pharmacological properties, including anticancer [56], anti-inflammatory [57], antioxidant [58], and anti-thrombotic activities and pleiotropic cardiovascular protective effects [59]. It also demonstrates therapeutic efficacy in Alzheimer’s disease [60]. Notably, kaempferol is being applied in cancer chemotherapy [61], holding significant research value in cancer treatment.

### 2.3. Dihydroflavones

Dihydroflavone derivatives include hesperidin, sakuranetin, isoliquiritigenin, naringenin, etc., with hesperidin being a paradigmatic example for its lipid-lowering activities. Hesperidin: Hesperidin is a flavonoid naturally abundant in citrus fruits such as *Citrus sinensis* and *Citrus reticulata* [62]. Structurally, it features the canonical tricyclic framework of flavonoids, where two benzene rings (A and B rings) are linked by a central three-carbon chain (C ring) to form a C6-C3-C6 skeleton. The C ring exists as a pyran ring with a single bond between C-2 and C-3 positions, adopting a cis configuration. Accumulating evidence indicates that hesperidin confers multifaceted benefits, including lipid-lowering [63], antioxidant [64], and anti-inflammatory [65] effects and neuroprotective effects against neurodegenerative disorders [31]. Therefore, daily intake of citrus fruits rich in hesperidin is associated with many health benefits.

### 2.4. Isoflavones

Isoflavone compounds include formononetin, isoliquiritin, maackiain, *soy isoflavones*, daidzein, calycosin, etc., among which formononetin is a representative example for its lipid-lowering effects. Formononetin: Also known as formononetin, it widely exists in leguminous plants (such as *Astragalus membranaceus*, *Pueraria lobata*, *Glycyrrhiza uralensis*, etc.), coffee beans, strawberries, and grapes [66,67,68,69,70]. Structurally, its C-ring serves as the bridge connecting the A and B rings, forming an unsaturated six-membered chromone ring. A critical feature is the double bond between C-2 and C-3 positions of the C-ring, endowing it with unsaturation—a defining structural characteristic of isoflavones. Studies from 1998 to 2019 have highlighted formononetin’s therapeutic potential in preventing and treating various diseases, including cancer, obesity, lipid-lowering, osteoarthritis, and neurodegenerative diseases [71,72].

### 2.5. Flavanols

Flavanol compounds include catechin, epicatechin, afzelechin, epigallocatechin, proanthocyanidins, trimeric flavanols, etc., with catechin being a representative example for its lipid-lowering effects. Catechin: It is a class of flavonoid compounds widely present in *Ampelopsis japonica*, *Lycium barbarum*, green tea, kiwifruit, etc. [73,74,75,76]. Its core structure is 2-phenylbenzopyran (flavanol), where two benzene rings (A and B rings) are linked by a three-carbon chain to form a pyran ring (C ring). The molecule contains multiple hydroxyl groups, endowing it with strong polarity and antioxidant properties. Studies have shown that catechin acts as a reactive oxygen species (ROS) scavenger and metal ion chelator, exerting antioxidant activity indirectly [77]. It also demonstrates the ability to delay muscle atrophy and enhance exercise capacity, thereby preventing, alleviating, delaying, or even treating muscle-related disorders caused by aging and diseases [78]. Additionally, catechin exhibits biological activities such as anticancer and lipid-lowering effects [79,80], holding promising potential for further development in pharmaceutical and food industries.

### 2.6. Chalcones

Chalcone compounds include licochalcone A, aurones, sulfuretin, carthamin, isoliquiritigenin A, morusin, etc., among which licochalcone A is a representative example for its lipid-lowering effects. Licochalcone A: Licochalcone A is predominantly found in *Glycyrrhiza uralensis* [81]. Its structure features two benzene rings linked by a three-carbon chain containing a central carbon–carbon double bond, forming an α,β-unsaturated ketone moiety. This unique conjugated system represents the key structural core for its biological activities. Owing to its beneficial effects, such as hypoglycemic regulation, lipid-lowering [82], and anti-allergic properties [83], licochalcone A has gained popularity in dietary applications.

### 2.7. Anthocyanidins

Anthocyanidin compounds include cyanidin-3-glucoside, anthocyanins, delphinidin, peonidin, malvidin, delphinidin-3-rutinoside, etc., among which cyanidin-3-glucoside is a representative example for its lipid-lowering effects. Cyanidin-3-glucoside: It is an important anthocyanin, mainly present in Artemisia argyi, mulberries, black wolfberries, purple cabbage, purple sweet potatoes, grapes, etc. [84,85,86]. Its molecular structure features a pyranidinium ring conjugated with two benzene rings, forming an extended conjugated system that dictates the compound’s color and chemical properties. Reported studies have shown that cyanidin-3-glucoside exhibits multiple biological activities, including anticancer [87] and lipid-lowering [88] activities and modulation of intestinal microbiota [89].

In conclusion, the seven classes of flavonoid compounds discussed above exhibit diverse biological activities, including lipid-lowering, anticancer, anti-inflammatory, and antioxidant effects, as summarized in Table 1. They play critical roles in the treatment of cancer, lipid metabolism disorders, and neurodegenerative diseases.

## 3. Mechanisms of Flavonoids in Treating Hyperlipidemia

Hyperlipidemia refers to elevated levels of total cholesterol (TC), triglycerides (TGs), or low-density lipoprotein cholesterol (LDL-C) in the blood, with pathological mechanisms involving pathophysiological processes across multiple systems. Flavonoids exert therapeutic effects against hyperlipidemia through multiple mechanisms, primarily by inhibiting lipid synthesis and absorption. The following summarizes the action mechanisms of the representative lipid-lowering flavonoids discussed above in treating hyperlipidemia in recent years.

### 3.1. Inhibition of Lipid Synthesis

#### 3.1.1. Inhibition of Triglyceride Synthesis

SREBP has three subtypes: SREBP-1a, SREBP-1c, and SREBP-2. Among them, SREBP-1a is responsible for the synthesis of lipids and cholesterol; SREBP-1c mainly promotes the transcription of genes related to fatty acid and TG synthesis; SREBP-2 is a regulatory factor that regulates the transcription of HMGCR (3-Hydroxy-3-Methylglutaryl-Coenzyme A Reductase) and the expression of LDL receptors [107]. SREBP needs to go through a series of biological processes, and it can only exert its transcriptional regulatory effect after being cleaved into active fragments and entering the nucleus. First, SREBP structurally binds to SCAP (SREBP cleavage-activating protein), and the formed SCAP-SREBP complex is retained on the endoplasmic reticulum membrane by INSIG1 (Insulin-Induced Gene 1). Under certain conditions, after SCAP is recognized by the COPΙΙ complex, the SCAP-SREBP complex dissociates from INSIG1, allowing SREBP to be transported from the endoplasmic reticulum to the Golgi apparatus under the escort of SCAP. In the Golgi apparatus, SREBP is cleaved by Site-1 protease (S1P) and Site-2 protease (S2P) to form active fragments, and nSREBP is formed to enter the nucleus [108]. Studies have found that apigenin can promote the expression level of INSIG1 by inhibiting miR-363-3p in cells and inhibit the expression of mature SREBP1 and its transcriptional effect on downstream lipid synthesis genes ACC1, FASN, and SCD, thereby regulating lipid synthesis [109]. Licochalcone A can also affect lipid synthesis and treat hyperlipidemia by inhibiting SERBP1 [110].

The PI3K-Akt signaling pathway is closely associated with lipid synthesis. Inhibition of PI3K-Akt pathway activation blocks insulin metabolism and suppresses cellular lipid synthesis [111,112]. PPARγ (peroxisome proliferator-activated receptor gamma) serves as a key adipogenic transcription factor, regulating the expression of triglyceride synthesis-related key enzymes such as fatty acid synthase (FAS) [113]. Studies have shown that flavonoids including apigenin, quercetin, and luteolin can effectively inhibit the PI3K-Akt signaling pathway and PPARγ protein expression, thereby regulating intracellular triglyceride levels [114].

The AKT/mTOR/SREBP1 signaling pathway represents an essential axis in lipid metabolism. In this pathway, AKT activates mTOR to enhance SREBP1 expression, thereby accelerating fatty acid production [115]. Through a combination of network pharmacology, metabolomics, molecular docking, and in vitro experiments, Tao Chen et al. validated that quercetin effectively treats hyperlipidemia by inhibiting the AKT/mTOR/SREBP1-mediated fatty acid synthesis pathway [116].

Adiponectin (Adipo), an adipokine, specifically binds to adiponectin receptor 1 (AdipoR1) and adiponectin receptor 2 (AdipoR2). This binding activates the AMPK and PPAR-α signaling pathways, thereby inhibiting fatty acid synthase enzymes (SREBP-1c and FASN) [117]. Additionally, flavonoids such as kaempferol and hesperidin have been reported to regulate triglycerides by targeting AKT [118,119]. Licochalcone A inhibits the PPARγ/SREBP1 axis to block adipocyte differentiation and lipogenesis [120], while cyanidin-3-O-β-glucoside mediates transcription of triglyceride lipases [121]. Quercetin activates the Adipo/AdipoR2 signaling pathway to reduce adipogenesis [122].

Lipoprotein lipase (LPL), which hydrolyzes triglycerides (TGs) into free fatty acids (FFAs), serves as a critical determinant of TG metabolism. Studies have shown that phosphorylation of AMP-activated protein kinase (pAMPK) inhibits LPL, making the pAMPK-LPL signaling pathway one of the key regulators of TG metabolism. Cyanidin-3-O-β-glucoside modulates TG metabolism by acting on the pAMPK-LPL signaling pathway [123].

#### 3.1.2. Inhibition of Cholesterol Synthesis

Endogenous cholesterol synthesis is one of the primary sources of cholesterol in the human body. The cholesterol biosynthesis pathway consists of approximately 33 steps, with HMGCR (3-Hydroxy-3-Methylglutaryl-Coenzyme A Reductase) serving as the rate-limiting enzyme, strictly regulating cholesterol homeostasis. INSIG-1 participates not only in the transcriptional and translational regulation of HMGCR but also in its ubiquitination-mediated degradation, dynamically modulating HMGCR activity based on intracellular cholesterol levels to maintain cholesterol homeostasis [124,125]. HMGCR activity can be inhibited to suppress cholesterol synthesis. For instance, AMPK phosphorylates HMGCR at the Ser872 site, thereby inhibiting its enzymatic activity [126]. Through transcriptomic analysis, Prasanth Puthanveetil et al. discovered that apigenin reduces cholesterol levels by inhibiting multiple transcripts involved in the cholesterol biosynthesis pathway, suggesting it may act by suppressing SREBP transcriptional targets [127]. Quercetin has also been reported to inhibit HMGCR activity to lower cholesterol levels [128]. 7-Dehydrocholesterol, a key intermediate in the cholesterol biosynthesis pathway, is converted to cholesterol under the catalysis of 7-dehydrocholesterol reductase (DHCR7) [129]. Cole Cochran leveraged untargeted metabolomics to prognosticate the therapeutic potential of apigenin, with in vitro experiments corroborating that apigenin downregulates 7-dehydrocholesterol—an intermediate in cholesterol biosynthesis—thus modulating lipid homeostasis [130].

### 3.2. Inhibition of Lipid Absorption

Niemann–Pick C1-like 1 (NPC1L1), predominantly expressed in intestinal epithelial cells, mediates endocytic uptake of exogenous cholesterol from the intestinal lumen [131]. Additionally, NPC1L1 contributes to the reabsorption of intestinal bile acids [132]. Reports have shown that luteolin exerts ezetimibe-mimetic effects by directly binding to NPC1L1, thereby inhibiting cholesterol absorption and treating hypercholesterolemia within a short timeframe [133]. Mari Nekohashi et al. demonstrated that both luteolin and quercetin suppress NPC1L1-mediated cholesterol uptake in Caco-2 and HEK293T cells [134]. Cholecystokinin (CCK), an appetite-regulating hormone, is commonly used to suppress appetite in obesity therapy. Hesperetin and hesperidin stimulate CCK release from enteroendocrine STC-1 cells, thereby reducing lipid intake [135].

### 3.3. Promotion of Cholesterol Uptake

Cholesterol uptake refers to the process by which cells acquire cholesterol from the bloodstream or the surrounding environment. Low-density lipoprotein (LDL) binds to LDL receptors (LDLRs) on the cell membrane, triggering endocytosis and the formation of endosomes. Under the acidic conditions within endosomes, LDL is released, while LDLRs recycle back to the cell surface for continuous LDL binding. Proprotein convertase subtilisin/kexin type 9 (PCSK9) binds to the LDLR/LDL complex, inhibiting LDLR release and promoting its lysosomal degradation, thereby disrupting LDLR recycling [136,137]. Majambu Mbikay et al. supplemented the high-fat diet of mice with quercetin-3-glucoside and observed that it suppressed PCSK9 expression and secretion, enhanced LDLR-mediated cholesterol uptake, and reversed diet-induced steatohepatitis, hyperlipidemia, and hyperinsulinemia. These beneficial effects were also replicated in pancreatic β-cells of the same mice [138]. Additionally, studies have reported that catechins can treat hyperlipidemia by modulating LDL uptake [139].

### 3.4. Promotion of Reverse Cholesterol Transport (RCT)

Reverse cholesterol transport (RCT) is the process by which cholesterol is transported from peripheral tissues (e.g., macrophages, vascular wall cells) to the liver via plasma lipoproteins, followed by excretion as bile acids or free cholesterol. The major steps include cholesterol efflux, HDL (high-density lipoprotein) maturation and transport, hepatic uptake and metabolism, and cholesterol excretion [140]. ATP-Binding Cassette A1 (ABCA1) facilitates cholesterol efflux, relieving lipid accumulation by accelerating cellular cholesterol efflux to promote macrophage RCT and HDL formation. Kun Ren et al. established an atherosclerosis model using ApoE−/−mice fed a high-fat diet and injected with LPS and then treated these mice with apigenin. The results showed that apigenin reduced serum TG, total cholesterol (CHO), and LDL levels while increasing HDL levels, indicating its role in regulating plasma lipoprotein cholesterol. In vitro experiments further revealed that apigenin significantly enhanced ABCA1-mediated cholesterol efflux and decreased cholesterol (CHO), free cholesterol (FC), and cholesteryl ester (CE) levels in macrophage-derived foam cells [141]. ABCG1 (ATP-Binding Cassette G1) shares similar functions with ABCA1, transporting cholesterol to mature HDL to form CE-rich HDL particles. Scavenger receptor class B type 1 (SRB1) primarily mediates hepatic uptake of HDL-derived CE, converting it to bile acids for excretion—the final step of RCT [142]. Liver X receptor α (LXRα), abundantly expressed in the liver, adipose tissue, and macrophages, directly promotes RCT by upregulating ABCA1, ABCG1, and CYP7A1 [143]. Luteolin modulates cholesterol levels by regulating LXRα, ABCG1, and SRB1 expression in HepG2 hepatocytes and livers of diet-induced obese mice [144]. Bile acid metabolism represents the final step of RCT, with bile acid biosynthesis accounting for ~40% of cholesterol elimination [145]. Cholesterol 7α-hydroxylase (CYP7A1), the rate-limiting enzyme in the classical bile acid synthesis pathway, is a promising target for hyperlipidemia therapy [146]. Dongliang Wang et al. demonstrated in vivo that cyanidin-3-O-glucoside upregulates hepatic cholesterol 7α-hydroxylase expression in high-fat diet-fed ApoE−/−male mice, reducing hypercholesterolemia [147].

### 3.5. Inhibition of Oxidative Stress

In a hyperlipidemic state, enhanced lipid peroxidation and reduced anti-lipid peroxidase activity occur. When oxygen radicals attack lipids, they disrupt hepatic lipid metabolism, triggering peroxidation reactions that generate excessive malondialdehyde (MDA). This further suppresses antioxidant enzyme activity, ultimately exacerbating oxidative stress and worsening hyperlipidemia [148]. Nrf2 (nuclear factor erythroid 2-related factor 2) resides primarily in the cytoplasm, chelated with Kelch-like Ech-associated protein (Keap1) and subjected to ubiquitination-mediated degradation. Upon activation by electrophilic agents like ROS (reactive oxygen species), Keap1 undergoes a conformational change, losing its ability to promote Nrf2 ubiquitination. This dissociates Keap1 from Nrf2, allowing Nrf2 accumulation and translocation to the nucleus, where it induces expression of antioxidant enzymes such as superoxide dismutase (SOD) and heme oxygenase-1 (HO-1) [149]. Jin-Ting Yang et al. found that luteolin not only reduced the myocardial lipid peroxidation product MDA but also enhanced nuclear translocation of Nrf2, upregulating downstream antioxidant genes NQO1 (quinone oxidoreductase 1) and HO-1 [150]. Hesperidin has been shown to protect against redox imbalance induced by hyperlipidemia in rats [151,152].

### 3.6. Promotion of Fatty Acid β-Oxidation

Fatty acid oxidation represents a critical metabolic process for energy production via fatty acid catabolism, playing a pivotal role in lipid homeostasis. Excessive fatty acid activation reduces β-oxidation, ultimately leading to hepatic lipid deposition. Fatty acid β-oxidation (FAβO), mediated by key catabolic enzymes, promotes β-oxidation of fatty acids to regulate lipid metabolism [153,154]. Jihan Sun et al. demonstrated that luteolin effectively lowers blood lipid levels in hyperlipidemic rats, enhances antioxidant enzyme activity, and reduces lipid peroxidation products. The mechanism may involve modulation of key enzymes regulating triglyceride levels, such as FAβO and fatty acid synthase (FAS) [155].

### 3.7. Regulation of Autophagy

Autophagic degradation of intracellular lipid droplets, termed lipophagy, decomposes triglycerides into free fatty acids (FFAs) within autolysosomes, thereby maintaining lipid homeostasis and preventing intracellular lipid accumulation [156]. Transcription factor EB (TFEB) serves as a key regulator of the autophagy–lysosome pathway, whose activity is inhibited by mTOR-mediated phosphorylation, leading to attenuated lipophagy [157]. AMPK activation phosphorylates and suppresses mTOR, counteracting this inhibitory effect [158]. Formononetin promotes the AMPK/TFEB pathway to ameliorate lipid accumulation in free fatty acid-stimulated HepG2 cells and primary mouse hepatocytes [159]. Zixuan Hu et al. treated atherosclerotic ApoE−/−mice with apigenin and observed reduced lipid levels. Western blot analysis of autophagy-related proteins demonstrated that apigenin may regulate lipid metabolism by activating autophagy in ApoE−/−mice [160].

### 3.8. Inhibition of Apoptosis

LOX-1 (lectin-like oxidized low-density lipoprotein receptor-1) serves as a specific receptor for oxidized low-density lipoprotein (ox-LDL), mediating ox-LDL uptake and intracellular signal transduction. This process promotes macrophage foam cell formation, induces lipid metabolism disorders, and contributes to diseases such as hyperlipidemia and atherosclerosis [161]. The Bcl-2 protein family plays a pivotal role in regulating apoptosis, with cellular apoptosis governed by the gene regulation of Bcl-2 and Bax. Consequently, most studies use the Bax/Bcl-2 gene ratio as an indicator of apoptotic status. Excessive hepatic lipid accumulation triggers hepatocyte apoptosis and injury, exacerbating hyperlipidemia and ultimately leading to liver dysfunction [162]. Qian Xu et al. demonstrated in vivo that apigenin effectively reduces serum lipid levels in hyperlipidemic rats, inhibits LOX-1 gene expression, and increases the Bcl-2/Bax ratio. These effects underlie its therapeutic actions against hyperlipidemia and prevention of atherosclerosis [163].

### 3.9. Inhibition of Inflammation

Activation of the NLRP3 (NOD-like receptor protein 3) inflammasome triggers the production of proinflammatory cytokines, such as interleukin (IL)-1β and IL-18. NLRP3 inflammasome involvement has been observed in cardiovascular diseases (CVDs), including hyperlipidemia, obesity, and atherosclerosis [164,165]. Zheng Lu et al. investigated the mechanism by which apigenin alleviates atherosclerosis and non-alcoholic fatty liver disease by inhibiting the NLRP3 inflammasome in mice. They found that apigenin reduces lipid accumulation by suppressing the NLRP3/NF-κB signaling pathway in HepG2 cells [166]. Other flavonoids, such as quercetin and hesperidin, exhibit similar effects [167,168].

### 3.10. Regulation of Gut Microbiota

Metabolomics and gut microbiomics have been widely applied to explore the mechanisms of drug therapy. The gut microbiota, composed of diverse microbial communities, produces metabolites that influence host metabolism. Emerging evidence has highlighted the critical role of the gut microbiota in the pathogenesis of hyperlipidemia [169,170]. Tongtong Wang et al. investigated the mechanism of quercetin in treating hyperlipidemia through plasma metabolomics and gut microbiome analysis. The study revealed that quercetin reduces lipid levels and improves liver function, potentially by regulating metabolism and the gut microbiota [171]. Tieqiao Wang explored the anti-obesity effects of kaempferol in high-fat diet (HFD)-fed mice and its impact on the gut microbiota, finding that kaempferol lowers blood lipid levels and modulates the gut microbiota to prevent obesity [172].

**Figure 1:**
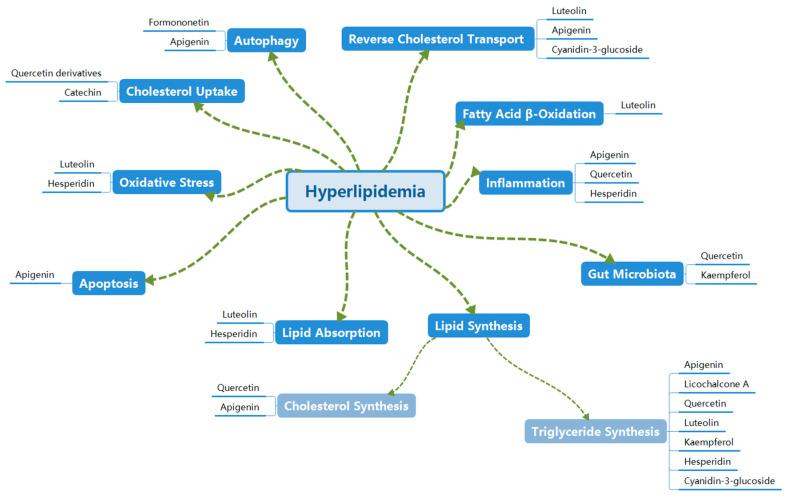
Mechanisms of flavonoids in treating hyperlipidemia.

In conclusion, extensive studies have identified ten major mechanisms by which flavonoids treat hyperlipidemia, as illustrated in Figure 1 and Table 2. These mechanisms include the following: inhibition of lipid synthesis, suppression of lipid absorption, promotion of cholesterol uptake, enhancement of reverse cholesterol transport, and inhibition of oxidative stress. Additionally, flavonoids modulate fatty acid β-oxidation, autophagy, apoptosis, inflammation, and the gut microbiota.

## 4. Bioavailability of Flavonoids

Apigenin is a type of flavonoid with poor oral bioavailability, low solubility, and high intestinal membrane permeability. It has low solubility in non-polar solvents (0.001–1.63 mg/mL in non-polar solvents) or high hydrophilicity (1.35 μg/mL in pure water). At pH 7.5, the maximum solubility in phosphate buffer is 2.16 μg/mL [173,174,175,176]. Apigenin is mainly transported through passive and active carrier-mediated saturation mechanisms in the duodenum and jejunum, as well as passive transport mechanisms in the ileum and colon. The aglycone apigenin can be rapidly absorbed in the perfused rat intestinal model. Studies have shown that after a single oral administration of radiolabeled apigenin, 51.0% of the radioactivity was recovered in urine, 12.0% in feces, 1.2% in blood, 0.4% in the kidneys, and 9.4% in the intestines within 10 days. Various results such as glucuronic acid and sulfated conjugate metabolites collectively indicate that apigenin has slow metabolism, slow absorption, and a slow elimination phase [173,177]. Due to the limitations of apigenin in absorption and bioavailability, there is a need to develop new carriers to improve its oral bioavailability, for example, by incorporating it into phospholipid preparations, nanosuspensions, polymeric nanoparticles, and nanocrystals [16].

Studies have reported that after oral administration of luteolin, the plasma luteolin level (3.04 ± 0.60 μg/mL) reaches a peak at 0.42 h, indicating that it can be rapidly absorbed. In addition, luteolin (60 mg/kg) is rapidly absorbed after intraperitoneal injection in rats. In contrast, its concentration peaks at 0.083 h (71.99 ± 11.04 μg/mL) with a longer half-life (3.2 ± 0.7 h), which shows that the absorption of luteolin varies with the administration method [178,179,180]. Luteolin has a strong metabolic effect, so its bioavailability is low after oral administration. The poor solubility and stability of luteolin are important obstacles to its clinical development. Therefore, a large number of studies have attempted to modify the structure of luteolin to improve its bioavailability, such as luteolin tetraphosphate and metal complexes of luteolin [181,182]. In terms of distribution, the distribution of luteolin may be affected by its binding to plasma proteins. In plasma, luteolin has a high affinity for human serum albumin, thus limiting its distribution in tissues with low protein-binding capacity [183]. Luteolin and its metabolites are mainly excreted and eliminated through the kidneys and biliary tract. Studies have reported that after oral administration, the half-life of luteolin is between 1 and 6 h, and most of the drug is excreted within 24 h. The main metabolite detected in urine is glucuronide, accounting for about 60% of the total excretion [184,185,186].

Quercetin is sensitive to pH and highly hydrophobic, and it can precipitate in gastric and intestinal juices, resulting in low bioavailability or bioaccessibility. Studies have shown that most of the biological activities of quercetin can only be achieved after quercetin is absorbed by the human body. However, the absorption of intact quercetin by healthy adults is relatively low [187,188]. In order to improve its bioavailability, most researchers have integrated nanoemulsion technology into preparations to solve the shortcomings of poor water solubility and poor oral absorption [189]. Quercetin biotransformation occurs through xenobiotic metabolism, which consists of three phases that act independently and/or additively to limit the absorption and accumulation of xenobiotics: phase I, modification; phase II, conjugation; and phase III, elimination. Its phase II metabolites include quercetin monoglucuronide, quercetin diglucuronide, quercetin sulfate, etc. The phase II metabolites of quercetin secreted from the small intestine reach the liver through the portal vein for further metabolism. Ingested quercetin can be rapidly excreted through feces and urine [190,191,192,193]. Finally, most quercetin-derived metabolites are identified as 3-hydroxyphenylacetic acid, benzoic acid, and hippuric acid [194].

Kaempferol is a highly polar glycoside, so its absorption is poor, and it is directly absorbed into the hepatic portal vein [57,195,196]. Studies have shown that after oral intake of kaempferol, kaempferol exists in plasma at nanomolar concentrations. Kaempferol found in chicory was also administered to eight healthy volunteers (246 mg kaempferol per kg of chicory). At 5.8 h after oral administration of chicory containing 8.65 mg kaempferol, high plasma concentrations of 100 nM kaempferol-3-glucoside (79%), 3-glucoside (14%), and 3-(6-malonyl)-glucoside (7%) were observed. Kaempferol metabolites are excreted through urine and bile. Proportions of 1.9% and 2.5% of the total dose are excreted through urine and bile, respectively [197].

Hesperidin has poor solubility and faces obstacles to its absorption in the gastrointestinal tract [198,199]. Unlike aglycones, hesperidin has poor membrane permeability and is mainly absorbed through the paracellular pathway [200,201]. Studies have shown that in the case of neurodegenerative diseases, hesperidin can cross the blood–brain barrier to reach the disease site and act directly where pathological processes occur [202].

Formononetin is rapidly absorbed orally, with the maximum plasma concentration occurring between 30 and 60 min and a half-life of about 2 h. It is metabolized by the liver through cytochrome P450 enzymes and phase II conjugation [203]. Studies have shown that after oral administration of formononetin to rats, the oral bioavailability of formononetin is 21.8%, and its absorption in the small intestine is better than that in the large intestine [204]. The plasma protein binding of formononetin is 93.61 ± 0.44% and 96.14 ± 0.15%. The bioavailability of free/relatively unchanged formononetin is only about 3%. In addition, it was found that the clearance rate of formononetin is as high as 5.13 L/h/kg [205,206]. At present, most studies use combined nano-formulations to enhance the bioavailability of formononetin for better application [207,208].

Catechin has low bioavailability due to its low gastrointestinal absorption rate [209]. Studies have shown that the concentration of catechin metabolites decreases sharply within 12 to 24 h after an experimental meal. Piskula et al. reported that the plasma epicatechin metabolite concentration decreased rapidly 8 h after oral administration of catechin in rats, indicating that catechin has a fast metabolic rate [210].

Although licochalcone A has many physiological functions, its wide application is limited due to its low solubility and bioavailability. There are few reports on the bioavailability of licochalcone A. Studies have reported that licochalcone A has an impact on P-glycoprotein (P-gp) as well as CYP3A4 and 2C9 activities. Licochalcone A inhibits CYP3A4 and CYP2C9 enzyme activities, with 50% inhibitory concentrations (IC50) of 2.0 and 0.1 μM, respectively [211]. In recent years, studies have also reported the development of self-microemulsifying drug delivery systems loaded with licochalcone A and licochalcone A-integrated casein–pectin nano-delivery systems to enhance its oral bioavailability and improve intestinal digestibility [212,213].

The bioavailability of cyanidin-3-glucoside is low. The small intestine is the site with the highest absorption rate of cyanidin-3-glucoside. It has been reported that after rats eat food rich in cyanidin-3-glucoside for 15 days, the content of C3G in the jejunum is 605 nmol/g, and the metabolites are combined with glucuronic acid [214]. Although the bioavailability of cyanidin-3-glucoside in the human body is too low, a large number of experiments have shown that cyanidin-3-glucoside can be detected in tissues and organs. For example, after rats were fed with cyanidin-3-glucoside-rich blackberries for 15 days, cyanidin-3-glucoside was found in many organs such as the liver, brain, jejunum, stomach, kidney, and even bones [215,216].

In conclusion, although various flavonoids have a variety of biological activities, their bioavailability is not ideal, which greatly limits their clinical application. Current studies have shown that methods such as combined nano-formulations can be used to improve their bioavailability for clinical practice.

## 5. Safety of Flavonoids

Currently, there are no reports on the toxic and side effects of apigenin on the human body. For example, Shoubaky et al. evaluated the acute toxicity of apigenin and found that there were no deaths or signs of toxicity in mice or rats with oral doses as high as 5000 mg/kg [217,218]. Luteolin has low toxicity, and due to its anticancer biological activity, it has a certain degree of toxic and side effects on cancer cells [219,220]. Ding S et al. found that luteolin can promote the apoptosis of hepatocellular carcinoma cells but has no effect on normal cells [221]. In addition, it has been reported that long-term and excessive use of luteolin can induce the consumption of glutathione, activate the metabolism of CYP450 (such as CYP3A), and mediate the formation of o-benzoquinone metabolites, thereby causing cytotoxicity in primary rat hepatocytes [222]. Adverse reactions related to quercetin only exist in a few cases. On the whole, quercetin has few toxic and side effects. Studies have shown that no adverse events occurred in subjects after continuous intake of 500 mg of quercetin per day for 4 to 8 weeks, 730 mg for 4 consecutive weeks, 1000 mg for 5 consecutive days, or at least 2 weeks or 12 consecutive weeks of such intake [223,224,225]. Through research, Ruscinc N et al. found that kaempferol can cause adverse reactions of vascularization in the chorioallantoic membrane, which indicates that kaempferol is a non-irritating compound [226]. In addition, because kaempferol can act as a pro-oxidant, some studies have found that kaempferol has mutagenic and genotoxic effects [57]. Some potential benefits of hesperidin use include its safety, non-accumulation, and limited side effects. In safety studies, it was administered to mice at a dose as high as 5%, and even with a relatively long administration time, no mutagenic, toxic, or carcinogenic effects were reported [227]. Formononetin showed mortality at an acute dose of 300 mg/kg and an LD50 of 103.6 mg/kg BW, with a NOAEL of 50 mg/kg BW. All other acute and subacute doses are safe when administered intraperitoneally [228]. In addition, studies have shown that acute and subacute toxicity tests of formononetin were conducted by intraperitoneal injection in mice. The results showed that the acute dose of FMN was 300 mg/kg, the LD50 was 103.6 mg/kg, and the NOAEL was 50 mg/kg. In the subacute toxicity test, there were no changes in the animals’ body weight, food intake, water intake, and behavior and no toxic effects or pathological damage to the organs. Therefore, it is proven that formononetin is safe and non-toxic and can be used for pharmacological and therapeutic purposes [228]. Through clinical trials, Matsuyama T et al. found that children who consumed beverages rich in catechins every day did not show any growth impairment due to catechin intake, but instead showed an effect of improving obesity [229]. Studies have shown that licochalcone A (197.1 μM) can scavenge 77.92% of free radicals. Licochalcone A at a concentration of 147.75 μM or higher can cause cytotoxicity in Chinese hamster ovary fibroblasts; however, there was no significant toxicity in biochemical markers and body weight [230]. Cyanidin-3-glucoside is generally similar to the above substances in terms of safety. Studies have reported that no serious adverse events occurred during the 2-week human study of black bean seed coat extract Cyanidin-3-glucoside, and there was only one minor adverse event, which may be unrelated to the consumption of black beans [231].

In conclusion, various flavonoid compounds generally show good safety, but there are some differences. Apigenin and hesperidin have outstanding safety with no reported obvious toxic or side effects, even at high doses or with long-term use; only a few cases or mild reactions have been reported for luteolin, cyanidin-3-glucoside, etc. Overall, the safety of flavonoids is relatively high, but the safety of each compound still needs to be explored systematically and scientifically.

## 6. Conclusions

Hyperlipidemia, a disorder characterized by abnormal lipid metabolism, stands as a key risk factor for cardiovascular diseases (CVDs), encompassing non-alcoholic fatty liver disease (NAFLD), obesity, and atherosclerosis. Despite the fact that flavonoids exert multifaceted therapeutic effects on hyperlipidemia, with the seven classes of flavonoid compounds discussed in this paper possessing diverse biological activities such as lipid-lowering, anticancer, anti-inflammatory, and antioxidant properties and playing significant roles in the treatment of cancer, lipid metabolism disorders, and neurodegenerative diseases, their underlying mechanisms remain incompletely elucidated. Currently, ten major mechanisms by which flavonoids treat hyperlipidemia have been identified, including inhibition of lipid synthesis, suppression of lipid absorption, promotion of cholesterol uptake, enhancement of reverse cholesterol transport, inhibition of oxidative stress, regulation of fatty acid β-oxidation, autophagy, apoptosis, inflammation, and modulation of the gut microbiota. Nevertheless, issues such as the specific mechanism by which apigenin downregulates 7-dehydrocholesterol reductase (DHCR7) and the precise pathway through which hesperidin protects hyperlipidemic rats against redox imbalance remain to be addressed. Furthermore, although flavonoids have been shown to treat hyperlipidemia through multiple mechanisms, their overall bioavailability is less than ideal. To tackle this problem of low bioavailability, most current studies have incorporated them into technologies such as nanosuspensions, polymeric nanoparticles, and nanocrystals to enhance their clinical applicability. In terms of safety, flavonoids exhibit low toxic and side effects and high safety. Although only a few cases of adverse reactions have been reported, the safety evaluation of flavonoids still requires systematic and scientific investigation. Statistical data reveal that CVDs cause 17.9 million deaths annually, accounting for 31% of global mortality, thus ranking as the leading cause of death worldwide [232]. Therefore, continuously deepening the exploration of the mechanisms underlying the improvement of hyperlipidemia by flavonoids holds great significance, providing a theoretical basis for the development of novel therapeutic approaches for hyperlipidemia.

## Figures and Tables

**Table 1:** Biological activities of representative flavonoids with lipid-lowering effects.

Category	HCAs	Structure	Source	Biological Activity/Application	Experimental Model	Dose Ranges	Ref.
Flavones	Apigenin (C_15_H_10_O_6_)	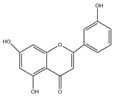	*Leonurus japonicus*, olive leaves, chamomile, parsley, celery, *Basella alba* L., artichoke, etc.	anti-inflammatory	in vitro	10, 20, 30 μM	[17]
				antioxidation	in vivo	20 mg/kg	[90]
				anticancer	in vivo	20–200 mg/kg	[19]
				anti-apoptosis	in vivo	10 mg/kg	[91]
				improve lipid metabolism	in vitro	40 μmol/L	[21]
				insulin resistance	in vivo	10 mg/kg	[92]
	Luteolin (C_15_H_10_O_7_)	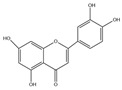	*Taraxacum mongolicum*, *Chrysanthemum morifolium*, lotus stems, *Lonicera japonica*, cabbage, celery, apples, oranges, pomegranates, lemons, etc.	including anticancer	in vivo	30 mg/kg	[93]
				lipid-lowering	in vitro	10–20 mm	[94]
				anti-inflammatory	in vitro	10 μm, 100 μm	[29]
				modulation of intestinal microbiota	in vivo	17.3 mg/mL	[30]
				immunoregulatory functions	in vivo	100 mg/kg	[95]
Flavonols	Quercetin (C_15_H_10_O_7_)	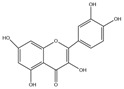	*Astragalus membranaceus*, *Trichosanthes kirilowii*, *Albizia julibrissin* flowers, and *Morus alba* leaves, as well as in fruits and vegetables including grapes, onions, carrots, and potatoes	ameliorating metabolic syndrome	in vivo	5, 10 mg/kg	[41]
				antibacterial effects	in vitro	1–8 mg/mL	[42]
				neuroprotection	in vivo	50, 100 mg/kg	[43]
				anti-ferroptotic activity	in vivo	25 mg/kg	[44]
				hypoglycemic	clinical	400 mg/kg	[41]
				anti-obesity properties	in vivo	240 mg/kg	[41]
				antirheumatic effects	in vitro	20, 40, 80 μmol/L	[46]
	Kaempferol (C_15_H_10_O_6_)	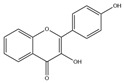	*Ginkgo biloba* leaves, *Carthamus tinctorius*, *Cnidium monnieri*, blueberry leaves, figs, legumes, cauliflower, cabbage, strawberries, tea, and tomatoes	including anticancer	in vitro	0, 25, 50, 75, 100 μM	[96]
				anti-inflammatory	in vivo	3, 5, 9 mg/kg	[97]
				antioxidant	in vivo	10 mg/kg	[98]
				Alzheimer’s disease	in vivo	10 mg/kg	[60]
Dihydroflavones	Hesperidin (C_28_H_34_O_15_)	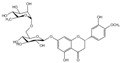	citrus fruits such as *Citrus sinensis* and *Citrus reticulata*	lipid-lowering	in vivo	150, 300 mg/kg	[99]
				antioxidant	in vivo	50 mg/kg	[64]
				anti-inflammatory	in vivo	200 mg/kg	[100]
				neuroprotective effects	in vivo	200 mg/kg	[101]
Isoflavones	Formononetin (C_16_H_12_O_4_)	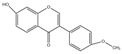	leguminous plants (such as *Astragalus membranaceus*, *Pueraria lobata*, *Glycyrrhiza uralensis*, etc.), coffee beans, strawberries, and grapes	including anticancer	in vivo	10 mg	[71]
				anti-obesity	clinical	30	[71]
				lipid-lowering	in vivo	10 mg/kg	[102]
				anti-osteoarthritis	in vitro	200 μM	[72]
				neuroprotective effects	in vitro	10 µM	[71]
Flavanols	Catechin (C_15_H_14_O_6_)	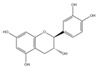	*Ampelopsis japonica*, *Lycium barbarum*, green tea, kiwifruit, etc.	antioxidation	in vitro	50 μM	[103]
				including anticancer	in vitro	25 μMol/L	[79]
				lipid-lowering	clinical	400 mg/d	[80]
Chalcones	Licochalcone A(C_21_H_22_O_4_)	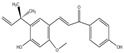	*Glycyrrhiza uralensis*	lower blood sugar	in vivo	100, 200, 300 mg/kg	[104]
				lipid-lowering	in vivo	5, 10 mg/kg	[105]
				anti-allergic properties	in vivo	20, 40, 80 mg/kg	[83]
Anthocyanidins	Cyanidin-3-glucoside (C_21_H_21_ClO_11_)	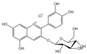	*Artemisia argyi*, mulberries, black wolfberries, purple cabbage, purple sweet potatoes, grapes, etc.	including anticancer	in vivo	5 mg/kg	[106]
				lipid-lowering	in vitro	100 μM	[88]
				modulation of intestinal microbiota	in vivo	250 mg/kg	[89]

**Table 2:** Molecular-level regulation of lipid metabolism by various flavonoids.

Category	HCAs	Mechanisms of Action	Classification of Mechanisms	Experimental Model	Dose Ranges	Ref.
Flavones	Apigenin (C_15_H_10_O_6_)	miR-363-3p↓, INSIG1↑, SREBP1, ACC1, FASN, SCD↓	Inhibition of Triglyceride Synthesis	HepG2 cells C57BL/6J mice	20, 40 μmol/L 150, 250 mg/kg	[109]
		PI3K, *p*-Akt, PPARγ↓		3T3-L1 preadipocytes	20, 40 μM	[114]
		SREBP2, HMGCR↓	Inhibition of Cholesterol Synthesis	mouse embryonic fibroblasts	50 μM	[127]
		7-Dehydrocholesterol, Xanthine↓		mouse embryonic fibroblasts	25 μM	[130]
		miR-33↓, ABCA1↑	Promotion of Cholesterol Uptake	RAW264.7 cells ApoE–/–mice	10, 20, 40 μM 10 mg/kg	[141]
		ULK1, UVRAG, beclin-1↑	Promote Autophagy	C57BL/6J mice	6.25, 12.5, 25 mg/kg	[160]
		LOX-1↑, Bcl-2, Bax↓	Inhibition of Apoptosis	Sprague Dawley rats	20, 40, 80 mg/kg	[163]
		NLRP3, NF-κB↓	Inhibition of Inflammation	HepG2 cells Ldlr−/−mice	25, 50 μM 5 mg/kg	[166]
	Luteolin (C_15_H_10_O_7_)	PI3K, *p*-Akt, PPARγ↓	Inhibition of Triglyceride Synthesis	3T3-L1 preadipocytes	10 μM	[114]
		NPC1L1↓	Inhibition of Lipid Absorption	Caco-2 cells	25–100 μM	[133,134]
		LXRα, ABCG1, SRB1↑	Promotion of Cholesterol Uptake	HepG2 cells C57BL/6J mice	10–50 μM	[144]
		Akt, Nrf2, NQO1, HO-1↑, mPTP↓	Inhibition of Oxidative Stress	Sprague Dawley rats	100 mg/kg	[150]
		FAβO↑, FAS↓	Promotion of Fatty Acid β-Oxidation	Sprague Dawley rats	50 mg/kg	[155]
Flavonols	Quercetin (C_15_H_10_O_7_)	PI3K, *p*-Akt, PPARγ↓	Inhibition of Triglyceride Synthesis	3T3-L1 preadipocytes	10 μM	[114]
		AKT, mTOR, SREBP1↓		Sprague Dawley rats	150 mg/kg	[116]
		Adipo, AdipoR2↑		3T3-L1 preadipocytes Swiss albino mice	5, 10, 20 μM 100 mg/kg	[122]
		HMGCR↓	Inhibition of Cholesterol Synthesis	C57BL/6J mice	5 mg/kg	[128]
		TLR4, NF-κB↓	Inhibition of Inflammation	Sprague Dawley rats	200 mg/kg	[138]
		beneficial bacteria↑, pathogenic bacteria↓, Firmicutes/Bacteroidetes↓	Regulation of Gut Microbiota	Sprague Dawley rats	10, 100, 200 mg/kg	[171]
	Kaempferol (C_15_H_10_O_6_)	AKT, SREBP1↓	Inhibition of Triglyceride Synthesis	HepG2, THP-1, cco2 cells	10, 20 μM	[118]
		relative abundance of Firmicutes↓, relative abundance of Bacteroidetes↑	Regulation of Gut Microbiota	C57BL/6J mice	200 mg/kg	[172]
Dihydroflavones	Hesperidin (C_28_H_34_O_15_)	*p*-Akt, NF-κB, Bcl-2↓	Inhibition of Triglyceride Synthesis	AML-1 human preadipocyte cell line	100, 500 µM	[119]
		STC-1, CCK↑	Inhibition of Lipid Absorption	3T3-L1 preadipocytes	0–1600 μg/mL	[135]
		LDL oxidation, PON-1↓	Inhibition of Oxidative Stress	Wistar rat	100 mg/kg	[151]
		IL-6↓; TNF-α↓	Inhibition of Inflammation	Sprague Dawley rats	50 mg/kg	[168]
Isoflavones	Formononetin (C_16_H_12_O_4_)	AMPK↑, TFEB↓	Promote Autophagy	HepG2 cells C57BL/6J mice	40 μM 100 mg/kg	[159]
Flavanols	Catechin (C_15_H_14_O_6_)	LDL-c uptake↑	Promotion of Cholesterol Uptake	HepG2 cells	4 μg/mL	[139]
Chalcones	Licochalcone A(C_21_H_22_O_4_)	LXRα, SREBP1↓	Inhibition of Triglyceride Synthesis	HepG2 cells	10 μg/mL	[110]
		PPARγ, SREBP1↓		3T3-L1 preadipocytes ICR mouse	5, 10 μM 5, 10 mg/kg	[120]
Anthocyanidins	Cyanidin-3-glucoside (C_21_H_21_ClO_11_)	O-glycosylation of FoxO1↑, FFAs, glycerol, ATGL↓	Inhibition of Triglyceride Synthesis	3T3-L1 preadipocytes	50 μM	[121]
		pAMPK↑, LPL↓		skeletal muscle cell, adipocyte KK-Ay mouse	10, 50, 100 µmol/L	[123]
		LXRα, CYP7A1↑	Promotion of Cholesterol Uptake	human aortic endothelial cell ApoE–/–mice	0.5, 5, 50 μM food (0.06% *w*/*w*)	[147]

## Data Availability

Data are contained within the article.
